# Expansion Microscopy for Cell Biology Analysis in Fungi

**DOI:** 10.3389/fmicb.2020.00574

**Published:** 2020-04-03

**Authors:** Ralph Götz, Sabine Panzer, Nora Trinks, Janna Eilts, Johannes Wagener, David Turrà, Antonio Di Pietro, Markus Sauer, Ulrich Terpitz

**Affiliations:** ^1^Department of Biotechnology and Biophysics, Theodor-Boveri-Institute, Biocenter, Julius-Maximilian-University, Würzburg, Germany; ^2^Institut für Hygiene und Mikrobiologie, Julius-Maximilian-University, Würzburg, Germany; ^3^Departamento de Genética, Universidad de Córdoba, Córdoba, Spain

**Keywords:** Expansion microscopy, fluorescence microscopy, fungi, *Aspergillus*, *Ustilago*, *Fusarium*, sporidia, hyphae

## Abstract

Super-resolution microscopy has evolved as a powerful method for subdiffraction-resolution fluorescence imaging of cells and cellular organelles, but requires sophisticated and expensive installations. Expansion microscopy (ExM), which is based on the physical expansion of the cellular structure of interest, provides a cheap alternative to bypass the diffraction limit and enable super-resolution imaging on a conventional fluorescence microscope. While ExM has shown impressive results for the magnified visualization of proteins and RNAs in cells and tissues, it has not yet been applied in fungi, mainly due to their complex cell wall. Here we developed a method that enables reliable isotropic expansion of ascomycetes and basidiomycetes upon treatment with cell wall degrading enzymes. Confocal laser scanning microscopy (CLSM) and structured illumination microscopy (SIM) images of 4.5-fold expanded sporidia of *Ustilago maydis* expressing fluorescent fungal rhodopsins and hyphae of *Fusarium oxysporum* or *Aspergillus fumigatus* expressing either histone H1-mCherry together with Lifeact-sGFP or mRFP targeted to mitochondria, revealed details of subcellular structures with an estimated spatial resolution of around 30 nm. ExM is thus well suited for cell biology studies in fungi on conventional fluorescence microscopes.

## Introduction

Fungi play important roles in human nutrition and well-being. These tiny organisms serve as biofactories in biotechnology and food industry, are essential for the biodegradation of complex organic compounds, but also act as highly destructive pathogens of plants, animals, and humans (Kendrick, 2011; Lange, 2014; Meyer et al., 2016; Cerimi et al., 2019). Recent studies estimate the number of fungal species to more than one million, many of which are specialized to specific ecological niches, thereby providing an arsenal of useful compounds (Blackwell, 2011; Hawksworth and Lücking, 2017; Naranjo-Ortiz and Gabaldón, 2019).

Microscopy allows to gain new insights at high spatial and temporal resolution into essential cellular processes such as protein localization, physiological activity, and growth dynamics (Hickey et al., 2004; Knaus et al., 2013; Chapuis et al., 2019). However, fluorescence microscopy of fungi is limited by the small size of their organelles, which is below the diffraction-limited resolution provided by conventional fluorescence microscopes. In addition, fungi tend to exhibit strong autofluorescence (Knaus et al., 2013), further complicating high-end fluorescence imaging.

To overcome these limitations, super-resolution microscopy has been developed and denoted substantial progress in the recent years (van de Linde et al., 2011; Coltharp and Xiao, 2012; Endesfelder and Heilemann, 2014; Dodgson et al., 2015; Heintzmann and Huser, 2017). Most applications, such as stimulated emission depletion (STED), photoactivated localization microscopy (PALM), *direct* stochastic optical reconstruction microscopy (*d*STORM) or structured illumination microscopy (SIM), adapt either the optical setup or exploit distinct photophysical properties of the sample, to allow image acquisition below the diffraction limit (Hohlbein et al., 2010; Chen et al., 2014). Such applications typically require guidance by experts and high financial investments to obtain the specialized microscopy setups.

By contrast, expansion microscopy (ExM) consists of expanding the whole cell including its subcellular structures in order to improve the resolution of fluorescence-based microscopy (Chen et al., 2015). Cells are fixed and immuno-stained, before amino groups are modified e.g., by glutaraldehyde (Chozinski et al., 2016), to enable incorporation of proteins, dyes and antibodies into a polyacrylamide hydrogel. After homogenization of the entire cellular context, e.g., by enzymatic treatment with proteinase K (Gao et al., 2017), the gel is isotropically expanded in water to uniformly extend the distances between fluorophores, allowing a lateral resolution of ∼60 nm by confocal microscopy (Chen et al., 2015). This simple idea was rapidly adapted by various laboratories, leading to the development of new protocols that allow expansion factors of up to 10× (Truckenbrodt et al., 2019) or even 20× by iterative expansion (Chang et al., 2017). Other protocols focus on preservation and isotropic expansion of ultrastructure (U-ExM) (Gambarotto et al., 2019) or on precise tuning of the expansion factor between 2 and 8 (ZOOM) (Park et al., 2019). Recently, ExM has been applied also to bacterial pathogens (Kunz et al., 2019) and to plants (Kao and Nodine, 2019), paving the way for new methodological approaches in these fields.

So far, ExM has not been used to visualize fungi. The application of ExM to fungi is challenging, since these organisms are surrounded by a complex cell wall that prevents uniform expansion of the cell content and largely differs in its composition from the cell walls of bacteria and plants (Latgé et al., 2017; Kang et al., 2018). Complete digestion of the cell wall is a prerequisite for the isotropic expansion of fungal cells. Since protoplasting protocols have been developed for a number of fungal species (Anderson and Millbank, 1966; Peberdy, 1979), we hypothesized that this approach could be applied to remove the cell wall after fixation and before embedding in the hydrogel to enable isotropic expansion.

The fungal cell wall consists of a complex mesh of components including chitin, β-1,3-glucan, α-1,3-glucan as well as different mannans and mannoproteins (Osherov and Yarden, 2010). Degradation of the cell wall is accomplished using combinations of lytic enzymes such as glucanex, which includes a cocktail of β-glucanases, cellulases, proteases and chitinases from *Trichoderma* species. Glucanex has been used for production of protoplasts in a number of fungi, including *Aspergillus spp.* (de Bekker et al., 2009) and *Fusarium spp.* (Ramamoorthy et al., 2015). Because cell wall composition varies strongly across fungal species and culture conditions (Reilly and Doering, 2010; Li et al., 2017), cell wall lysis protocols need to be carefully optimized for each condition and fungal strain.

In this study we show that both ascomycete and basidiomycete fungi are suitable for ExM after treatment with cell wall lytic enzymes. Isotropically expanded fungal cells (∼4.5-fold) were submitted to CLSM and SIM (Gustafsson et al., 2008) for super-resolution fluorescence imaging. We imaged *Ustilago maydis* sporidia expressing a fluorescent version of the membrane rhodopsin UmOps1 (Panzer et al., 2019) as well as *Fusarium oxysporum* hyphae expressing histone H1-mCherry (Ruiz-Roldan et al., 2010) and the F-actin marker Lifeact-sGFP (Fernández-Ábalos et al., 1998; Riedl et al., 2008). Moreover, we show that the ExM protocol can be used successfully for super-resolution fluorescence imaging of the clinically relevant human pathogen *Aspergillus fumigatus*.

## Materials and Methods

### Fungal Strains / Cultivation

The *F. oxysporum f. sp. lycopersici* race 2 strain 4287 (FGSC 9935) was used in all experiments. The *F. oxysporum* mutant constitutively expressing histone H1 fused to mCherry red fluorescent protein (H1-mCherry) was previously described (Ruiz-Roldan et al., 2010). To obtain a *F. oxysporum* strain simultaneously expressing both H1-mCherry and the Lifeact-sGFP fluorescent reporter for F-actin visualization (Fernández-Ábalos et al., 1998; Riedl et al., 2008), protoplasts of the previously obtained H1-mCherry strain were co-transformed with a hygromycin resistance cassette plus a P_*gpdA*_::LifeAct-sGFP linear fragment (for details see Supplementary Text S1 and Supplementary Table S1), as previously described (Di Pietro et al., 2001; López-Berges et al., 2012). For microconidia production, cultures were grown in potato dextrose broth (PDB; Sigma P6685) at 28°C and 120 rpm for 4–7 days. Microconidia were filtered through a custom-made cotton filter system, harvested by centrifugation and washed twice with pure water.

The *U. maydis* strains expressing opsin1 fused to enhanced green fluorescent protein (eGFP) were described in detail before (Panzer et al., 2019) and derived from the wild type isolate FB1 (Banuett and Herskowitz, 1989). Either the strain FB1 ΔUmOps1 P_*crg*_::UmOps1-eGFP K1 or FB1 P_*crg*_::UmOps1-eGFP KA was used for ExM experiments. *U. maydis* sporidia were grown as described before (Panzer et al., 2019). Sporidia were grown in PDB for 15–24 h at 28°C and 100 rpm, harvested by centrifugation (4000 × *g*, 3 min), washed once in ddH_2_O and resuspended in ddH_2_O to a final density of 6.5 x 10^5^ sporidia/mL. If required, expression of UmOps1-eGFP was induced by treatment with arabinose-containing induction medium (Panzer et al., 2019).

The non-homologous end joining-deficient *A. fumigatus* strain AfS35 (Krappmann et al., 2006; Wagener et al., 2008) was transformed with plasmids pYZ011 and pYZ012. pYZ012 harbors a phleomycine resistance cassette and encodes a mitochondria-targeted red fluorescent protein (RFP) fusion protein that consists of the N-terminal part (52 amino acids) of *Aspergillus niger* citrate synthase followed by mRFP1 under control of the *Aspergillus nidulans gpdA* promoter. pYZ011 harbors a pyrithiamine resistance cassette and encodes *A. fumigatus* AFUA_1G10040 including 230 bp of its promoter region, followed without stop codon by the coding sequence of a GFP (S65T) (Heim et al., 1995), FPbase ID: B6J33 derivative with the following modifications: M1_S2insV, E235_K238delinsSCTSKISRPRETW. The strain was cultivated on solid Aspergillus minimal medium (AMM) (Hill and Kafer, 2001) in T75 tissue culture flasks (Sarstedt) in order to avoid uncontrolled spreading of the hydrophobic fungal spores. Spores were harvested by submerging them with PBS and resuspending them by means of glass beads.

### Enzymatic Digestion of the Cell Wall and Staining

Fungal spores and sporidia were seeded onto poly-D-lysine (PDL)-coated coverslips in 4-well tissue culture plates (800 μL/well). While sporidia were investigated after 30 min of sedimentation, conidia were allowed to germinate in the respective culture medium for 18 h (*A. fumigatus*) or 14–19 h (*F. oxysporum*). If indicated, fungal cells were incubated for 5–10 min in 0.5 μM mCling-Atto643 fluorescent dye dissolved in nutrient media to stain the membrane. After three washing steps with PBS the samples were either directly fixed in 4% formaldehyde and 0.25% glutaraldehyde for 15 min (standard procedure) or, as in case of microtubule detection in *U. maydis* sporidia, treated according to the protocol of Michie et al. (2017). Briefly, sporidia were prefixed and permeabilized for 1 min in prewarmed cytoskeleton buffer (10 mM MES buffer pH 6.1, 150 mM NaCl, 5 mM EGTA, 5 mM glucose and 5 mM MgCl_2_) containing additionally 0.25% Triton X-100 and 0.3% glutaraldehyde and finally fixed for 10 min in cytoskeleton buffer supplied with 2% glutaraldehyde. For quenching of autofluorescence, fixation was followed by a 7 min incubation in 0.1% NaBH_4_. After consecutive washing, cell walls were digested for 1 h at RT with a cell wall lytic enzyme solution, based on an enzymatic mix (0.1 g lysing enzyme of *T. harzianum*, 0.25 g driselase, and 0.5 mg chitinase dissolved in 10 ml 0.7 M NaCl) that was used for the generation of *Fusarium fujikuroi* protoplasts before (García-Martínez et al., 2015). The enzymatic solution was either directly used in the experiments or stored at −80°C for later use. For treatment of young germlings and *U. maydis* sporidia the enzyme solution was diluted in a ratio of 1:5 with 0.7 M NaCl. In case additional antibody-staining was required, the samples were blocked for 30 min in 5% BSA / 0.25% Triton X-100 and subsequently incubated with the primary anti-α-tubulin antibody (abcam, ab18251) for 1 h. After washing, the samples were incubated for another hour in the corresponding secondary antibody (Alexa 488-label, Thermo-fisher, A11008 or ATTO647N-label, Sigma, 40839) resolved in blocking solution and washed with PBS. All samples were instantly processed for ExM.

### Expansion

Immediately before gelation, as previously published (Chozinski et al., 2016; Kunz et al., 2019), the samples were incubated for 10 min with 0.25% glutaraldehyde and washed with PBS. Thereafter, a droplet of the monomer solution [8.625% sodium acrylate (Sigma, 408220), 2.5% acrylamide (Sigma, A9926), 0.15% N,N′-methylenbisacrylamide (Sigma, A9926), 2 M NaCl (Sigma, S5886), 1 × PBS and 0.2% freshly added ammonium persulfate (APS, Sigma, A3678) and tetramethylethylenediamine (TEMED, Sigma, T7024)] was prepared on parafilm in a humid Petri dish. Using tweezers, the coverslip with the attached fungi was then transferred upside-down on the gelation droplet. The sample was allowed to gelate for at least 1 h at RT in the closed dish. To ensure isotropic expansion, samples were homogenized (Gao et al., 2017) in digestion buffer [50 mM Tris pH 8.0, 1 mM EDTA (Sigma, ED2P), 0.5% Triton X-100 (Thermo Fisher, 28314), and 0.8 M guanidine HCl (Sigma, 50933)] supplied with 8 U/ml proteinase K (Thermo Fisher, AM2548) for 1 h to overnight. This step is required to reduce the cohesion of the fixed proteins while most cellular compounds are washed out the gel. At the same time the majority of the fluorophores remain attached to the polymer (Tillberg et al., 2016). Digested samples were expanded in ddH_2_O for 3–4 h. The water was changed every hour until the size of the gel did not increase any more. Expanded gels were stored at 4°C until use. The expansion factor was determined by both the diameter of the fungi as well as by the gel size before and after expansion. Imaging was performed in PDL-coated chambers (Merck, 734-2055) to immobilize the gels.

### Fluorescence Microscopy

Imaging was performed on a confocal inverted system (Zeiss LSM700) or on SIM system (Zeiss ELYRA S.1 SR-SIM) equipped with an 63x oil (used for unexpanded samples) and a 63x water-immersion objective (used for ExM samples; C-Apochromat, 63 x 1.2 NA, Zeiss, 441777-9970). The water objective was necessary to provide sufficient working distance to be able to image the expanded samples. Unexpanded CLSM images were captured using the ideal pixel sizes provided by the software (between 740 x 740 and 856 x 856 pixels) and a pixel dwell time between 1.58 μs and 4.24 μs using laser powers ranging between 1.5 and 10%. The expanded samples were then imaged again with the optimum pixel size and pixel dwell time ranging from 6.30 μs to 8.43 μs, using laser powers between 10 and 26% with lasers of 488 nm, 555 nm and 639 nm. The pinhole was adjusted to 1 airy unit and the photomultiplier was set to 700. SIM images were reconstructed with the ZEN image processing platform of the SIM module, with a fixed pixel size of 31 nm. Laser power ranged between 8 and 25% using laser of 488 nm, 561 nm, and 642 nm with integration times between 100 ms and 300 ms. For final image processing, Imaris 8.4.1 and FIJI 1.51 (Schindelin et al., 2012) were used.

## Results

### Degradation of the Cell Wall With Lytic Enzymes Enables Expansion of Filamentous Fungi and Sporidia

Expansion microscopy relies on isotropic expansion of all cellular structures during gel swelling. Therefore, it is crucial that the fungal cell wall is completely digested to enable the uniform movement of labeled proteins and/or fluorophores during the swelling process.

**FIGURE 1:**
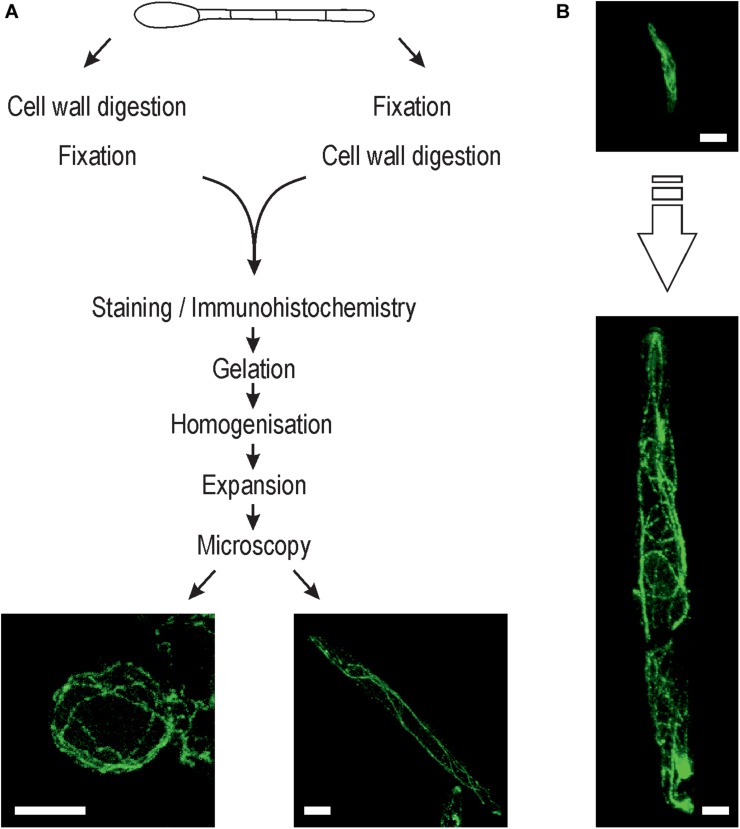
Expansion microscopy of fungi. **(A)** Schematic overview showing the steps involved in the expansion protocol for fungi. Inset: Representative ExM-CLSM images of an *Ustilago maydis* protoplast (left) and sporidium (right) stained with anti-α-tubulin antibody (ATTO647N). Cell wall digestion was always performed after initial fixation to ensure preservation of the structural information of sporidia and hyphae. **(B)** Typical *U. maydis* sporidium before (top) and after (bottom) expansion at the same scale. Scale bars, 10 μm **(A)** and 5 μm **(B)**.

The fungal cell wall contains rigid polymers to maintain turgor pressure and to avoid undesired cell swelling in fungi, and thus needs to be removed to enable uniform expansion of the fungal cell in the polyelectrolyte hydrogel. We successfully degraded the cell wall in *F. oxysporum* and *A. fumigatus* germlings and *U. maydis* sporidia using a mixture of glucanex, driselase and chitinase. Complete removal of cell wall material was confirmed by labeling with the chitin-specific dye calcofluor white (Supplementary Figure S1). The used expansion protocol was based on previously established protocols (Chozinski et al., 2016; Kunz et al., 2019). We noted that the time point of cell wall digestion within the protocol workflow significantly affected the morphology obtained after the expansion process. Cell wall lysis before fixation resulted in the generation of protoplasts with spherical shape, leading to impaired distribution of the subcellular structures as compared to untreated cells (Figure 1). Protoplasts might still be useful for imaging depending on the scientific question addressed. On the other hand, when cell wall digestion was performed after fixation, the original shape of the hyphae and sporidia was preserved (Figure 1).

In the three fungal species tested, cell wall removal was successfully accomplished after fixation with either 4% formaldehyde or 4% formaldehyde + 0.25% glutaraldehyde. As reported previously (Li et al., 2017; Wu and Chou, 2019), the germination time of *F. oxysporum* conidia had a significant impact on the outcome of cell wall digestion, with longer germination times resulting in incomplete cell wall digestion that impaired the expansion process (Supplementary Figure S2).

After digestion of the cell wall, fungal cells were expanded by a factor of 4.53 ± 0.08 (*n* = 9) as determined from the gel dimensions before and after expansion. In expanded samples, details of subcellular structures were visualized by CLSM and SIM as described in detail in the following sections.

### Expansion of *Ustilago maydis* Enhances Resolution of the Cytoskeleton and Membrane Protein Distribution

**FIGURE 2:**
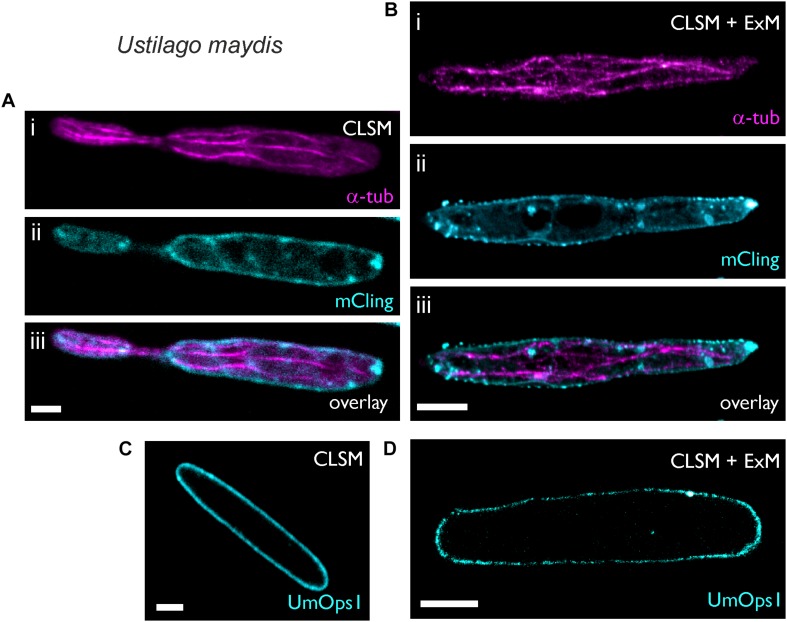
Expansion microscopy of *Ustilago maydis* sporidia. Sporidia were imaged after fixation, either before **(A,C)** or after 4.6-fold expansion **(B,D)**. **(A,B)** Sporidia were stained for 5 min with the (i) membrane stain mCling and then (ii) immunostained with a primary antibody against α-tubulin. Maximum intensity projection of the secondary antibody’s Alexa488-signal. For better visualization the mCling signal is only shown from a middle level. **(C,D)** Expression of the fungal rhodopsin UmOps1 fused to eGFP in the plasma membrane of the sporidia. Scale bars, 2 μm **(A,C)** and 10 μm **(B,D)**.

*Ustilago maydis* is a dimorphic fungus, which undergoes a morphological transition from the yeast form to the filamentous form (Kahmann and Kämper, 2004; Vollmeister et al., 2012). The haploid sporidia represent the yeast form, which proliferates by budding. Untreated sporidia have a diameter of 2.4 ± 0.35 μm (*n* = 26) and exhibit subcellular structures that are below the diffraction limit of resolution and thus cannot be resolved by conventional microscopy (Figure 2A). Here we found that the cell wall of sporidia was easily removed by treatment with the lytic enzyme cocktail, which allowed expansion by a factor of 4.6 resulting in a diameter of 11 ± 1.08 μm (*n* = 9). Confocal images of expanded cells accurately visualized the cytoskeleton and plasma membrane (Figure 2B). Most importantly, membrane vesicles were clearly visible in the expanded samples (Figure 2B). These results demonstrate that ExM can isotropically expand intracellular structures of fungi.

Next, we investigated whether or not membrane proteins in *U. maydis* can be visualized in expanded samples. To test this, we used a *U. maydis* strain heterologously expressing UmOps1-eGFP, a microbial rhodopsin that was recently shown to act as a green-light driven proton pump (Panzer et al., 2019). Localization of UmOps1 in the plasma membrane could be observed before (Figure 2C) and after expansion (Figure 2D). In the latter case, the fluorescent membrane protein was isotropically expanded 4.5-fold, visualizing the shape of the expanded sporidium, and the fluorophore density decreased 91-fold. As a consequence, in some areas the fluorescence appeared weak or non-homogeneously labeled in the expanded images (Figure 2D).

### ExM of the Ascomycete *Fusarium oxysporum* Reveals Structural Information

The soil-inhabiting ascomycete *F. oxysporum* causes vascular wilt disease in more than a 100 different crop species and has been reported as an opportunistic human pathogen. Similar to *U. maydis*, the plasma membrane of *F. oxysporum* can be stained with mCling dye, which stably remains in membranes after fixation (Revelo and Rizzoli, 2016).

**FIGURE 3:**
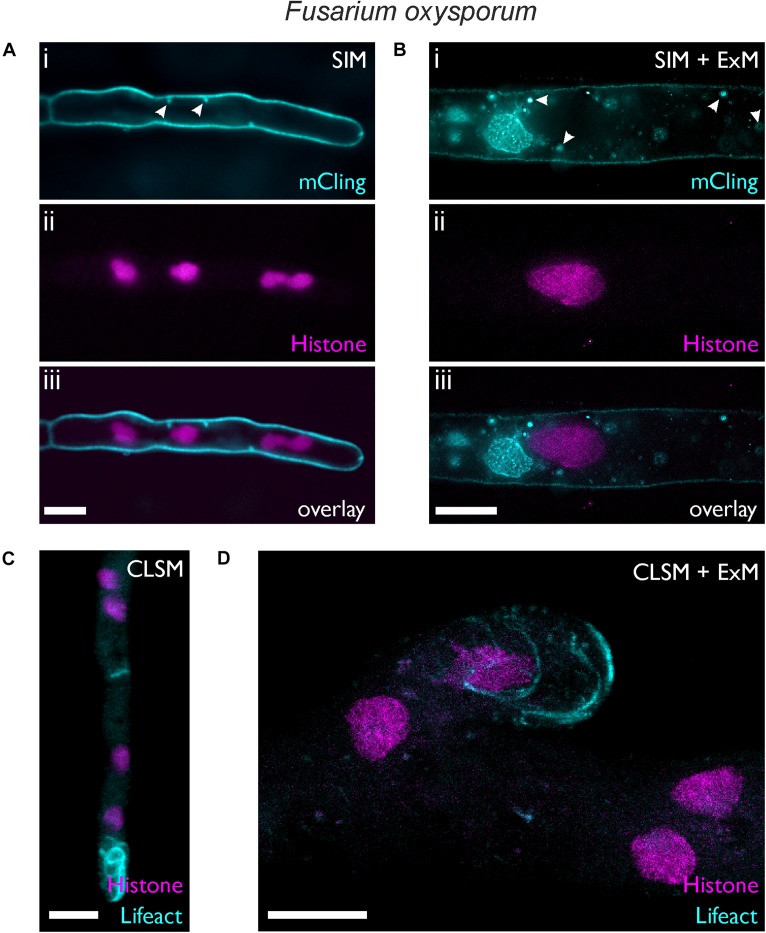
Expansion microscopy of *F. oxysporum*. Images were taken before **(A,C)** and after expansion **(B,D)**. **(A,B)** SIM images showing hyphae of a strain expressing histone H1-mCherry (i) stained 5 min **(A)** or 10 min **(B)** with mCling. (ii) Membrane-bound vesicles are highlighted by white arrows. **(C,D)** CLSM maximum intensity profile images of a strain expressing lifeact-sGFP and histone H1-mCherry. Scale bars, 5 μm **(A,C)** and 10 μm **(B,D)**.

We used a *F. oxysporum* strain expressing histone H1 labeled with mCherry (Ruiz-Roldan et al., 2010). With a hyphal diameter of 2.89 ± 0.49 μm (*n* = 17), the standard SIM image provides only limited information on the intracellular structure (Figure 3A). In contrast, imaging of the expanded fungal hypha with an average diameter of 12.73 ± 0.71 μm (*n* = 4), clearly revealed different membrane embedded organelles and vesicles in addition to the histone-H1-labeled nuclei (Figure 3B). Depending on the time of staining, mCling tends to stain also the intracellular membranes as seen in the expanded *F. oxysporum* hypha that was incubated with mCling for 10 min (Figure 3B).

By using the *F. oxysporum* histone H1-mCherry strain as genetic background, we generated a strain expressing both histone H1-mCherry and the F-actin reporter Lifeact-sGFP (Riedl et al., 2008). Figures 3C,D show the distribution of filamentous actin together with the nuclei. The formation of actin filaments at the hyphal tip was visible both in the expanded and non-expanded sample, but the distinct actin cables (bundles of actin filaments) could not be resolved well in the non-expanded sample due to the small diameter of *F. oxysporum* hyphae, a situation similar to that reported in *A. nidulans* (Bergs et al., 2016). The superior resolution provided by ExM allowed observation of the actin cables, similar to *N. crassa* hyphae which naturally exhibit a much larger hyphal diameter (Berepiki et al., 2010). Within the nucleus, regions with higher and others with lower fluorescence intensity were visible, possibly reflecting differences in histone density as described in living cells of mammals (Nozaki et al., 2017) and plants (Rutowicz et al., 2018) PREPRINT. Such differences may appear more pronounced due to the diluted fluorescence intensity after expansion.

### ExM Reveals the Distribution of Mitochondria in Hyphae of the Mold *Aspergillus fumigatus*

**FIGURE 4:**
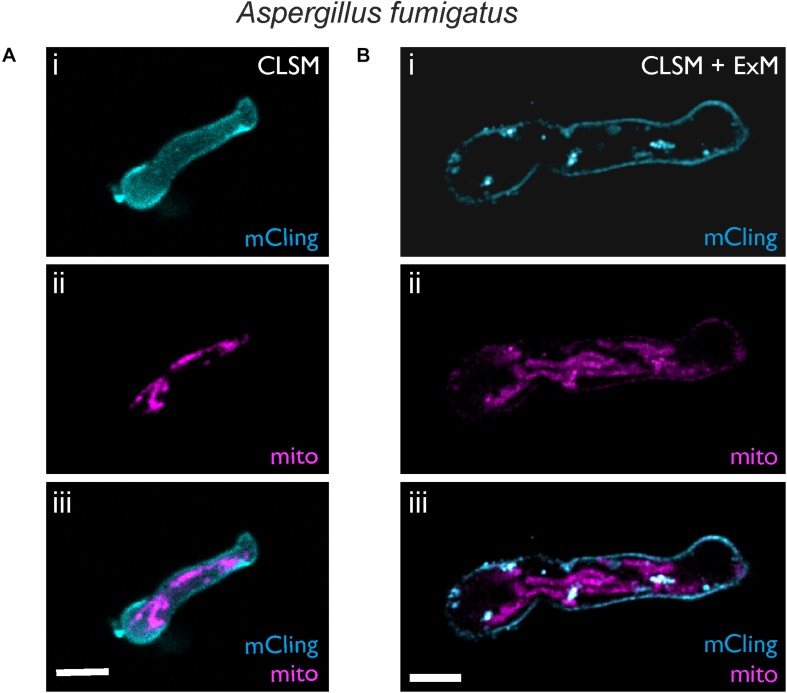
Expansion microscopy of *A. fumigatus* with mRFP1-labeled mitochondria. CLSM images before **(A)** and after **(B)** expansion are shown. Samples were stained with mCling to label the plasma membrane (i, cyan). Mitochondria were visualized by the fluorescence of mRFP1 (magenta, ii). In the overlay (iii) after expansion the shape of the mitochondria and the plasma membrane becomes clearly resolved using standard CLSM. Scale bars, 5 μm **(A)** and 10 μm **(B)**.

*Aspergillus fumigatus*, a mold with worldwide distribution, produces masses of highly hydrophobic conidia that are ubiquitous in the air and can cause life-threatening invasive aspergillosis when inhaled by immunocompromised patients. We used ExM to visualize an *A. fumigatus* strain expressing mRFP fused to the N-terminus of citrate synthase, thus conferring localization in the mitochondria. Cell wall digestion of *A. fumigatus* hyphae was obtained within the first 18 h of germination (Figure 4). The membrane dye mCling was successfully used to visualize the shape of hyphae. Typically, *A. fumigatus* hyphae exhibited mean diameters of 2.43 ± 0.27 μm (*n* = 9), which increased 4.4-fold to 10.72 ± 0.69 μm (*n* = 17) after digestion and expansion.

While the shape of single mitochondria could not be distinguished well before expansion (Figure 4A), it was clearly resolved in the expanded hyphae (Figure 4B). However, fluorescence intensity of mRFP1 was low, possibly due to the proteinase treatment. The use of different proteinases or antibodies as an alternative to fluorescent proteins could further enhance the fluorescence signal.

## Discussion

In recent years, fluorescence microscopy has seen a boost in technical advances, with a number of new technologies mainly directed at circumventing the diffraction limit of optical resolution (Coltharp and Xiao, 2012; Schermelleh et al., 2019). Major drawbacks of these technologies are the high financial investment and the technical expertise required for running such setups. Therefore, super-resolution microscopy installations are often limited to core units of research institutes.

The recent introduction of ExM provides an attractive alternative that can be implemented in almost every laboratory with access to conventional fluorescence microscopy. Instead of using sophisticated optical or computational upgrades, in ExM the sample itself is physically enlarged to enhance the resolution of the specimen that can be obtained with a standard fluorescence microscope. Assuming a spatial resolution limit of 250 nm, ExM increases the effective resolution to about 60 nm (Chen et al., 2015). Using SIM on expanded samples, one can expect further increase of the spatial resolution to ∼30 nm.

The aim of this work was to transfer the concept of ExM to fungi, since many fungal research groups lack the infrastructure required for super-resolution microscopy. Our results show that ExM is generally suitable for studying fungal cell biology. Structures from all three fungal species used here, including sporidia of the basidiomycete *U. maydis* and hyphae of the ascomycetes *A. fumigatus* and *F. oxysporum*, could be expanded using similar protocols with only minor modifications in culture times and enzymatic cell wall treatment. Preparation of samples for ExM requires complete removal of the cell wall, since in the rare cases of incomplete cell wall digestion the expansion proceeded non-isotropically (Supplementary Figures S1, S2). Therefore, not all of the available protocols for protoplast generation in fungal species can be directly transferred to meet the specific requirements of ExM. While partial digestion of the hyphal cell wall can be sufficient to release protoplasts to the environment, ExM requires complete removal of the cell wall to avoid artifacts. Here we used a combination of lysing enzyme of *T. harzianum*, driselase, and chitinase, a cocktail conferring β-glucanase, cellulase, protease, chitinase, laminarinase, and xylanase activities, that was successfully used for protoplast generation in *F. fujikuroi* (García-Martínez et al., 2015). Our protocol allowed efficient expansion of young germlings of less than 12 or 18 h in *F. oxysporum* and *A. fumigatus*, respectively. After longer growth periods the composition of the cell wall appeared to change, making it more resistant to the deconstruction by lytic enzymes. By contrast, in *U. maydis* sporidia cell wall digestion was successful at any culture age due to its yeast-like growth. The importance of the culture conditions in cell wall digestion for protoplast generation has been reported previously in filamentous fungi (Reilly and Doering, 2010; Li et al., 2017) and yeasts (Terpitz et al., 2012).

Recent investigations of the cell wall composition of *A. fumigatus* suggest an important contribution of α-1,3-glucan to the rigid inner domain (Kang et al., 2018). Since α-1,3-glucanase is not commercially available, it was not part of our lytic enzyme cocktail. Further optimization of the lytic enzyme mixture could in the long-term allow ExM of older hyphae, or hyphae growing under challenging conditions. This may be of particular interest for the visualization fungal hyphae attacked by immune cells (Park and Mehrad, 2009), where the fungus is pre-germinated and maintained in co-cultures for up to 12 h (Ziegler et al., 2017).

After embedding the digested hyphae in the gel, our protocol followed the classical expansion procedure successfully used for mammalian cells, tissue sections, and bacteria (Chozinski et al., 2016; Kunz et al., 2019). These protocols provided isotropic expansion of hyphae and sporidia by a factor of 4.6 (*U. maydis*) or 4.4 (*F. oxysporum* and *A. fumigatus*), calculated from the cell size measured before and after expansion. In agreement with this, the macroscopic analysis of gel expansion revealed an expansion factor of 4.53 ± 0.08 (*n* = 9).

Importantly, our finding that intracellular vesicles maintain the circular shape after expansion (Figure 3), provides clear evidence that this protocol produces isotropic expansion of fungal cell. This was further confirmed by the conserved shape of the plasma membrane before and after expansion, as visualized with mCling (Figures 2–4). In the three fungal systems used here, ExM strongly improved visualization of the shape of organelles. For example, differences in the histone H1 distribution were observed in expanded nuclei of *F. oxysporum* hyphae, that are similar as reported previously for nuclei in mammalian cells (Nozaki et al., 2017) while such information was not resolved in unexpanded hyphae with the same microscopical settings (Figure 3). Similarly, membrane-surrounded vesicles were visible as hollow spheres after ExM, but not in the unexpanded sample. Finally, the morphology of mitochondria was detectable in expanded *A. fumigatus* hyphae, but not in the unexpanded samples (Figure 4).

The additional subdiffractional information gained by ExM also has some costs. Expansion results in a decrease of fluorophore density, leading to a reduced fluorescence signal. For example, eGFP-labeled rhodopsin occasionally appeared devastated or non-homogeneously distributed in the ExM image, due to a strong reduction of fluorophore density after expansion by almost two orders of magnitude with an expansion factor of 4.5. Nevertheless, the spotty distribution may reflect the natural situation, since a similar pattern was observed in correlative fluorescence and electron microscopy images of 250 nm-sections of *F. fujikuroi* expressing the rhodopsin CarO-eYFP (unpublished data).

Expansion microscopy imposes a limitation in the type of fluorophores that can be used, because common carbocyanines such as Cy5 or Alexa Fluor 647 become deteriorated during the gelation (Tillberg et al., 2016). In addition, the treatment of the hyphae embedded in the gel with proteinase K may result in partial degradation of the fluorescent proteins, leading to further reduction of fluorescence intensity. Both drawbacks can be addressed by using more efficient fluorophores or immunohistochemistry to enhance the fluorescence signal. On the positive side, the removal of cell wall content leads to a reduction of the associated autofluorescence, which can be intense in fungi such as *A. fumigatus* (Ziegler et al., 2017). Since autofluorescence often interferes with the visualization of fluorescent dyes or proteins, loss of autofluorescence concomitant with preservation of the fluorescence signal can lead to an increased signal to noise ratio and thus improve visualization of fluorescent structures.

In conclusion, our results demonstrate that ExM is readily applicable to fungi after successful treatment with cell wall degrading enzymes. In our experiments, ExM allowed the visualization of ultrastructural information that is below the resolution of a conventional fluorescence microscope. Since ExM can be used advantageously in all labs with access to conventional fluorescence microscopes, the optimized ExM protocol will be of general interest to the broad field of fungal research.

## Data Availability Statement

The raw data supporting the conclusion of this article will be made available by the authors, without undue reservation, to any qualified researcher.

## Author Contributions

RG, SP, and JE performed the expansion experiments. SP and NT optimized fungal culture. RG, SP, and JE acquired microscopic images. JW generated the *A. fumigatus* strain, DT and AD the *F. oxysporum* strain. MS and UT conceived this study and supervised the experiments. UT wrote the manuscript and designed the figures. All authors provided discussions and contributed to the manuscript.

## Conflict of Interest

The authors declare that the research was conducted in the absence of any commercial or financial relationships that could be construed as a potential conflict of interest. The reviewer FE declared a past supervisory role with one of the authors JW to the handling editor.
