# Supplementary material for: Expansion Microscopy for Cell Biology Analysis in Fungi
Source: Front Microbiol. 2020 Apr 3;11:574. doi: 10.3389/fmicb.2020.00574 (PMC7147297; doi:10.3389/fmicb.2020.00574)
Supplement: Supplementary file 1 [file Data_Sheet_1.pdf]

Supplementary Material

Supplementary Text 1

The P_{gpdA}::Lifeact-sGFP construct consisting of the 17 aa Lifeact peptide C-terminally fused to sGFP via a poly-glycine linker under the control of the strong *Aspergillus nidulans gpdA* promoter was obtained by PCR fusing the partially overlapping P_{gpdA} and Lifeact-sGFP amplicons with the primer pair Gpda15nest & Actgfprn (Supplementary Table 1). To generate the two overlapping fragments, the P_{gpdA} and Lifeact-GFP fragments were obtained by PCR amplification from the pAN8-1 (Mattern et al., 1988) and pAB221 (Berepiki et al., 2010) plasmids with the use of the primer pairs Gpda15B & Gpda9 and Actgfpfor & Actgfpr, respectively. Selected hygromycin-resistant transformants were purified by monoconidial isolation and analysed by fluorescence microscopy.

Supplementary Table 1. Primers used for synthesis of the P_{gpdA}::LifeAct-sGFP construct

Actgfpfor	CCGCTTGAGCAGACATCACAATGGGCGTCGCTGACCTCA
Actgfpr	TCAATTTCGCCTCGTTCGTCA
Actgfprn	TCATGCAGTTGGCTAAGGTTG
Gpda9	TGTGATGTCTGCTCAAGCGG
Gpda15B	GGATCCCGAGACCTAATACAGCCCCT
Gpda15nest	ACTCAAATCGACTTCAGCAACA

Literature

Berepiki, A., Lichius, A., Shoji, J.-Y., Tilsner, J., Read, N. D. (2010). F-Actin Dynamics in *Neurospora crassa*. *Eukaryotic Cell*, *9*(4), 547–557. https://doi.org/10.1128/EC.00253-09

Mattern, I., Punt, P., Hondel, C. V. den. (1988). A vector for *Aspergillus* transformation conferring phleomycin resistance. *Fungal Genetics Reports*, *35*(1), 25. https://doi.org/10.4148/1941-4765.1533

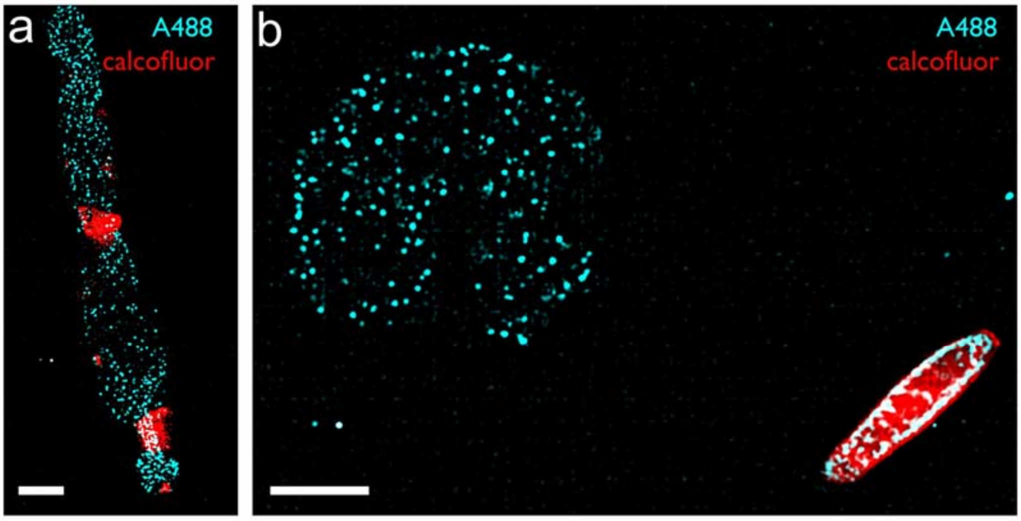

Supplementary Figure S1. Incomplete digestion of cell wall components leads to non-isotropic expansion, exemplified with *U. maydis* sporidia (a) and protoplasts (b). Calcofluor (red) was used to stain remaining fragments of the cell wall and visualized by SIM. Cell shape was visualised by unspecific binding of an alexa-488-labelled antibody. Note that regions stained in red display impaired isotropic expansion due to cell wall residues. Scale bars, 5 µm.

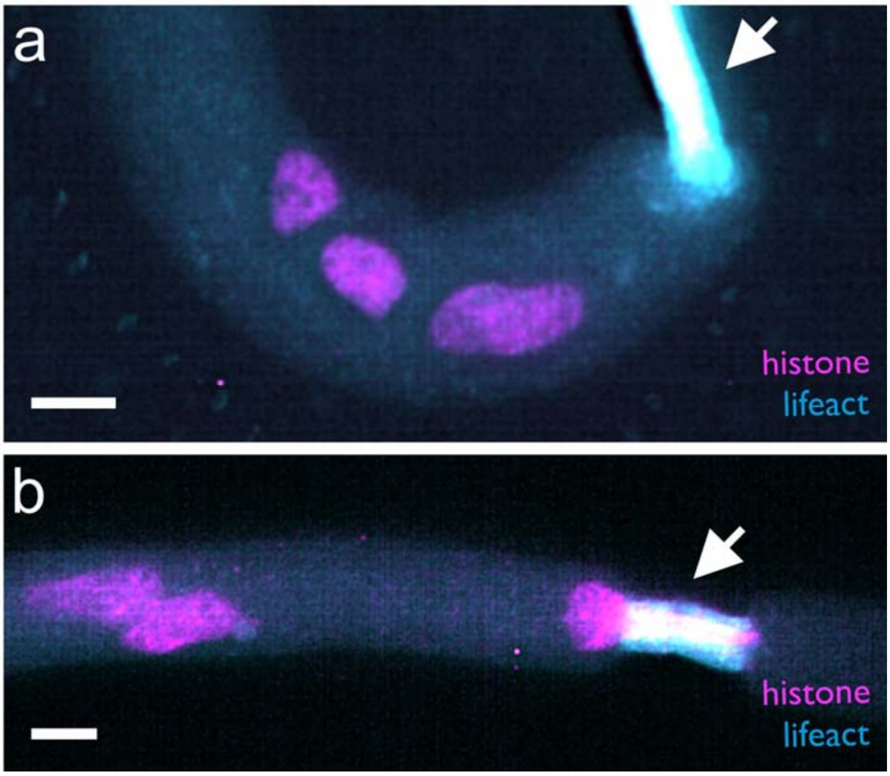

Supplementary Figure S2. CLSM images showing incomplete expansion of *F. oxysporum* hyphae expressing histone H1-mCherry and Lifeact-sGFP. Regions showing incomplete expansion and enrichment of actin fluorescence are highlighted by arrows. Scale bars, 5 µm.
